# Supplementary figures and images for: Circulating Tumor DNA-Based Disease Monitoring of Patients with Locally Advanced Esophageal Cancer
Source: Cancers (Basel). 2022 Sep 11;14(18):4417. doi: 10.3390/cancers14184417 (PMC9497103; doi:10.3390/cancers14184417)

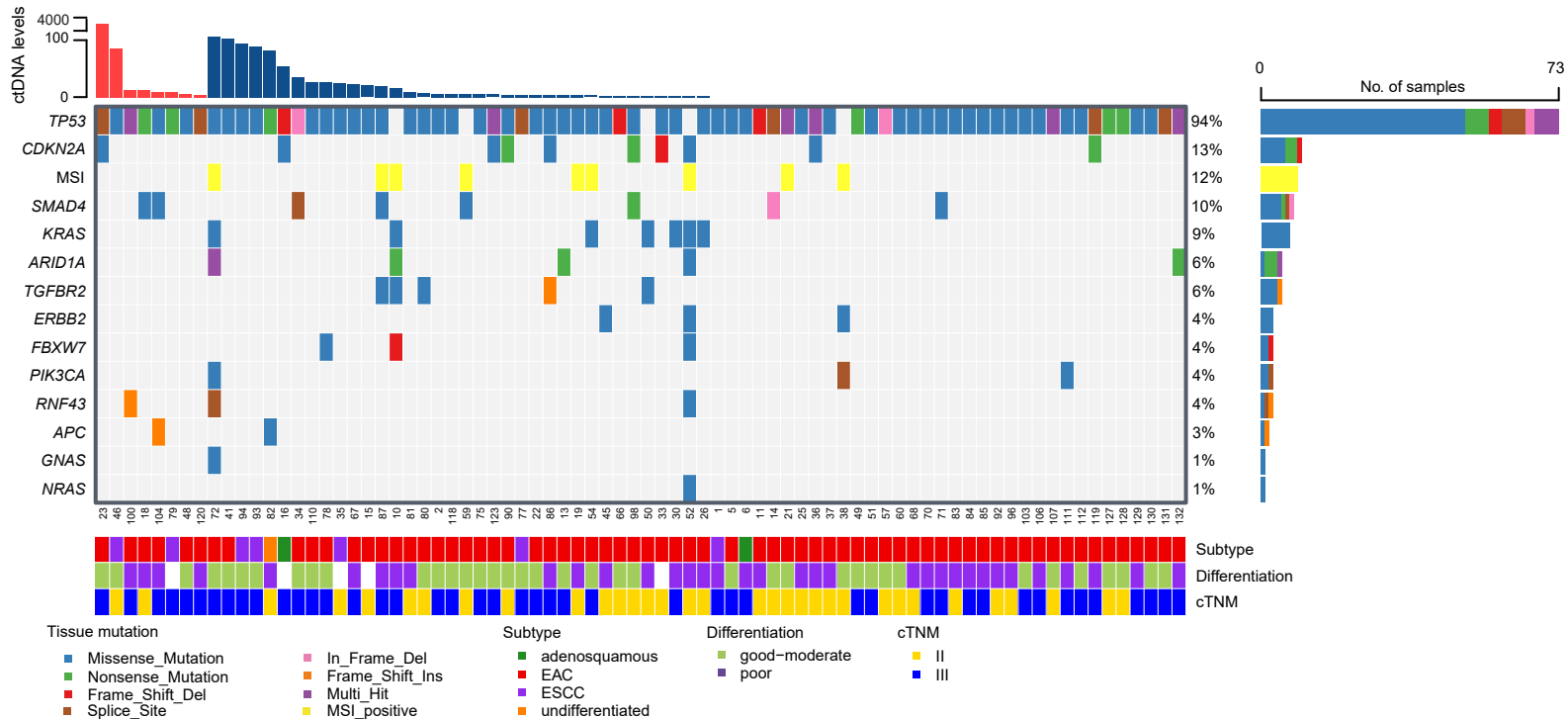

Supplement: Supplementary file 1 [file cancers-14-04417-s001.zip › Supplemental figure S1.pdf]

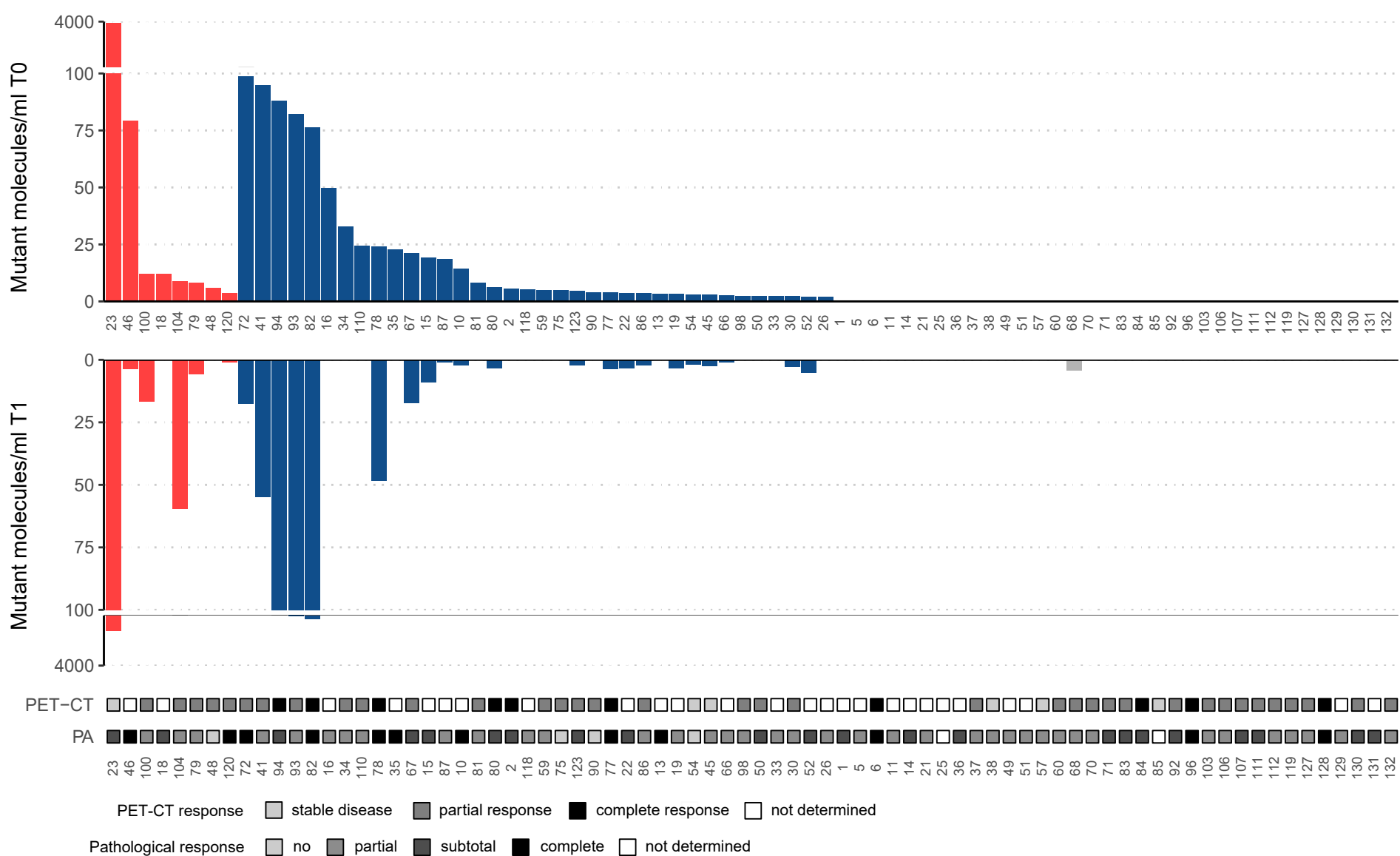

Supplement: Supplementary file 1 [file cancers-14-04417-s001.zip › Supplemental figure S2.pdf]
